# How Fast Is Recovery of Impaired Glucose Tolerance after 21-Day Bed Rest (NUC Study) in Healthy Adults?

**DOI:** 10.1155/2014/803083

**Published:** 2014-03-11

**Authors:** Martina Heer, Natalie Baecker, Stephan Wnendt, Annelie Fischer, Gianni Biolo, Petra Frings-Meuthen

**Affiliations:** ^1^Department of Nutrition and Food Science-Nutrition Physiology, University of Bonn, 53115 Bonn, Germany; ^2^Profil Neuss GmbH, 41460 Neuss, Germany; ^3^German Aerospace Center (DLR), Institute of Aerospace Medicine, 51147 Cologne, Germany; ^4^MLM Medical Labs GmbH, 41066 Monchengladbach, Germany; ^5^Division of Internal Medicine, University of Trieste, 34127 Trieste, Italy

## Abstract

*Aim*. We hypothesized that 4 days of normal daily activity after 21 days of experimental bed rest (BR) will not reverse BR induced impaired glucose tolerance. *Design*. Glucose tolerance of seven male, healthy, untrained test subjects (age: 27.6 (3.3) years (mean (SD)); body mass: 78.6 (6.4) kg; height: 1.81 (0.04) m; VO_2_ max: 39.5 (5.4) ml/kg body mass/min) was studied. They stayed twice in the metabolic ward (crossover design), 21 days in bed and 7 days before and after BR each. Oral glucose tolerance tests were applied before, on day 21 of BR, and 5 and 14 days after BR. *Results*. On day 21 of BR, AUC_120 min_ of glucose concentration was increased by 28.8 (5.2)% and AUC_120 min_ of insulin by 35.9 (10.2)% (glucose: *P* < 0.001; insulin: *P* = 0.02). Fourteen days after BR, AUC_120 min_ of serum insulin concentrations returned to pre-bed-rest concentrations (*P* = 0.352) and AUC_120 min_ of glucose was still higher (*P* = 0.038). Insulin resistance did not change, but sensitivity index was reduced during BR (*P* = 0.005). *Conclusion*. Four days of light physical workload does not compensate inactivity induced impaired glucose tolerance. An individually tailored and intensified training regime is mandatory in patients being in bed rest to get back to normal glucose metabolism in a reasonable time frame.

## 1. Introduction

Exercise is one of the very promising measures to reduce blood glucose concentrations potentially by enhancing insulin-mediated glucose uptake [1]. On the other hand, physical inactivity promotes high blood pressure, decreases insulin sensitivity and muscle mass and strength, and supports obesity. Thereby, even 3 days of bed rest is sufficient in healthy subjects to induce impaired glucose tolerance following an oral glucose tolerance test (OGTT) [2–5]. But not only extreme inactivity like bed rest induces impaired glucose tolerance in young healthy people. Thyfault and Booth summarized recently that “decreased frequency of breaks from sitting, as well as longer total duration of daily sitting, acutely lowers insulin sensitivity which would chronically increase metabolic risk” [6], which is supported by recent surveys in USA [7] and Australia [8].

But more and more people in the western world, even young people, live a rather sedentary life style. Data derived from the 2003-2004 National Health and Nutritional Examination survey (NHANES) demonstrate that only 8% of adolescents (12–19 years) and less than 5% of adults (≥20 years) follow the recommendation of 30 to 60 min of exercise per day [9]. Taken into account that skeletal muscle loss occurs as early as five days of disuse and that extreme inactivity such as in-bed rest is often a component of critical illness it would be worthwhile to examine how long it takes until the pre-bed-rest state of glucose tolerance is restored when keeping a moderate exercise level after bed rest. In this context, however, it would also be important which exercise level an individual was adapted to before assuming total inactivity like bed rest [1, 2].

Another impact aside from the sedentary lifestyle on insulin action is energy balance. Stephens et al. [10] have shown that in normal weight, metabolically healthy, fit test subjects when inactivity (sitting) is combined with positive energy balance (energy surplus) insulin action is reduced by 39%, while sitting during balanced energy intake reduces insulin action only by 18% compared to insulin action while being active. They concluded that “strategies to limit daily sitting may reduce metabolic risk disease” [10]. A more pronounced effect of lower physical activity compared to positive energy balance is also reviewed by Mann et al. [1].

We were able to add, as a pilot experiment, glucose tolerance measurements before, during, and after a very complex study, nutritional countermeasures (NUC) study, investigating the effects of potassium bicarbonate supplementation during head-down tilt bed rest on markers of bone resorption. In our add-on experiment we hypothesized that four days of normal daily activity in the ward after 21 days of experimental bed rest (BR) will not reverse BR induced impaired glucose tolerance in sedentary subjects.

## 2. Material and Methods

### 2.1. Study Design

Seven metabolically healthy, male, sedentary subjects (mean age: 27.6 (SD 3.3) years, mean body mass: 78.6 (SD 6.4) kg, mean body mass index (BMI): 24.1 (SD 1.9) kg/m^2^, and VO_2_ max: mL/kg body mass/min: 39.5 (SD 5.4)), no first degree relatives being diabetic, were included in the “nutrition countermeasures” (NUC) bed-rest study at the German Aerospace Center in Cologne, Germany, in 2010. Approval for the study was obtained from the Ethical Committee of the Aerztekammer Nordrhein, Duesseldorf, Germany, and the study was conducted in accordance with the Declaration of Helsinki. Volunteers gave their written informed consent after receiving detailed information about the study protocol and the resulting risks. Inclusion criteria for subjects were passing medical and psychological tests. Exclusion criteria were any history of hypertension, diabetes, obesity, rheumatism, hyperlipidemia, hepatic disease, bone disease, physical exercise more than four times per week, smoking, consumption of drugs, or alcohol excess. Moreover subjects needed to have negative results of a thrombophilia screening panel (AT III, S-Akt, Lupus-PTT, ferritin, Factor V Leiden, Factor IV, and Factor II) since they would be exposed to immobilization during the study.

The study was a randomized crossover design in two campaigns separated by 154 days (last day of the first bed-rest campaign and the first bed-rest day of the second campaign). The subjects were confined to the metabolic ward 7 days before to 6 days after the respective bed-rest period. Each campaign consisted of seven days of pre-bed-rest baseline data collection (BDC-7 to BDC-1), 21 days of strict six-degree head-down tilt bed rest (HDT1 to HDT21), and six days of post-bed-rest recovery (R+0 to R+5). Reambulation occurred in the morning of R+0 between 9.00 and 11.00 am. Subjects also returned to the facility on R+14 for follow-up assessment. During the bed-rest period, subjects were kept in bed for 24 h and were not allowed to elevate their head >30° from the horizontal. The lying position was confirmed by constant video monitoring. All daily activities (such as showering, weighing, reading, and watching television) and all lavatory activities were carried out in this position. During the non-bed-rest phases the subjects were allowed to walk around in the ward without performing any physical exercise. For the whole duration of the study a study nurse made sure they adhered to the study rules. The study was approved by the ethical committee of the Aerztekammer Nordrhein, Duesseldorf, Germany, and subjects gave their informed written consent.

As mentioned in the introduction, the primary outcome parameters of the NUC study were bone resorption markers in 24 h urine. However, we were able to include OGTTs before and during inactivity and after reassuming. These tests were carried out in fasting state in the mornings of BDC-4, HDT21, R+5, and R+14.

### 2.2. Dietary Standardization

For the first campaign, subjects were randomized to the standard nutrition group or to the standard nutrition plus potassium bicarbonate group. In the second campaign the subject was obligated to participate in the other group as part of the crossover design. All subjects received a standardized nutrition of 1.2 g protein per kg body mass per day, 1000–1200 mg calcium per day, 3.0–5.0 g potassium (plus 90 mmol per day in the potassium bicarbonate group) per day, 2.1–2.3 mmol sodium per kg body mass per day, 1000 IU vitamin D_3_ per day, and 50 mL water per kg body mass per day. The individually tailored energy intake (total energy expenditure) was calculated by multiplying resting metabolic rate measured by indirect calorimetry with the Deltatrac device (Deltatrac II MBH 200 metabolic monitor, Datex-Ohmeda) by a physical activity level of 1.4 during the ambulatory phase (for light physical activity) and by 1.1 during bed rest and then adding 10% of total energy expenditure for thermogenesis. The daily diet was also constant for fat (28–31% of total energy expenditure) and carbohydrates (50–55% of total energy expenditure). All other nutrients met the recommended dietary allowances [11]. The menu composition of each experiment day of the respective study campaigns was identical in order to avoid any impact of variable glycemic indices on plasma glucose and insulin concentrations. Potential renal acid load (PRAL) calculated according to Remer and Manz [12] was −53.2 mEq/d in the first campaign and −51.9 mEq/d in the second campaign. When subjects participated in the potassium bicarbonate group, they drank 90 mmol (3 × 30 mmol during main meals) potassium bicarbonate as a soluble tablet dissolved in 200 mL tap water daily during the bed-rest phase, whereas the control group received 200 mL tap water.

Power analysis for this study was based on the primary outcome measure bone resorption marker in 24 h urine and not the parameter of the presented data. However, retrospective power analyses for the effect of bed rest on glucose and insulin concentration at an alpha level of 0.05 revealed a power of 100% for both, glucose and insulin concentrations.

### 2.3. Sample and Data Collection and Analyses

In the morning (7 am) on days BDC-4, HDT21, R+5 (all in-house), and R+14 an oral glucose tolerance test (OGTT) was applied using 75 g of glucose dissolved in 200 mL tap water, while subjects stayed in supine position. Blood was drawn from the antecubital vein just before (0 min) and 30, 90, and 120 min after glucose application for analyses of serum glucose and insulin concentrations. These time points were chosen to cover a 2-hour period with the blood volume we were allowed to draw. For C-reactive protein (CRP), total cholesterol, and triglycerides blood was only drawn in fasting conditions on days BDC-6, BDC-2, R+2, and R+5. Whole blood was immediately centrifuged and then aliquoted. Serum aliquots were stored at −20°C for later analysis.

Serum glucose was analyzed by the hexokinase/glucose-6-phosphate dehydrogenase method (Glucose HK, Roche Diagnostics, Mannheim, Germany) using an automated analyzer (INTEGRA 400/700/800).

Serum insulin was analyzed via an insulin ECLIA (Electrochemiluminescence Immunoassay) from Roche Diagnostics, Mannheim, Germany, using a Roche Hitachi Modular E170 lab automate. C-reactive protein, cholesterol, and triglyceride concentrations have been analyzed in the BDC and the recovery period using commercial reagents from Roche Diagnostics, Mannheim, Germany, applied on a Roche Hitachi Modular P800 lab automate.

Areas under the serum concentration curve (AUC) of glucose and insulin concentrations were calculated according to the trapezoidal rule. Insulin resistance was calculated by the homeostatic model assessment (HOMA-IR2) according to Levy et al. [13]. Insulin sensitivity index (ISI_composite_) was calculated according to the equation of Matsuda and DeFronzo [14]. We also calculated hepatic insulin resistance (HIR) by using the equations of Abdul-Ghani et al. [15].

Body mass was determined daily after emptying bladder and before breakfast on a sensitive weight scale (Sartorius, type DVM 5703 MP 8-1). Body composition was analyzed by DXA (HOLOGIC Discovery A) (upgraded model of HOLOGIC 4500W) on day BDC-5 and day R+1.

In the BDC phase of each campaign and on R+28 of the second campaign, subjects completed a questionnaire on habitual physical activity [16]. This physical activity questionnaire has been validated previously [16] and assesses occupational (e.g., time spent standing, sitting, lifting heavy items, and so forth at work), sport-related (i.e., types of sports played, how frequently, and how long), and leisure-time (e.g., extent of television viewing) physical activities. Based on the evaluation of these questionnaires and as presented in the publication of Belavý et al. [17] post-bed-rest activity was not different from pre-bed-rest activity.

### 2.4. Statistics

Data on subjects' characteristics are presented as means ± SD and all other values as means ± SEM. The data were analyzed using repeated measures ANOVA techniques (SAS, Version 9.2). The case of log-normal distribution of the data was tested. In case of no normal distribution, logarithmic data was used for further analyses. Differences between study days were tested by ANOVA. Statistical significance was defined as *P* < 0.05.

## 3. Results

**Figure 1:**
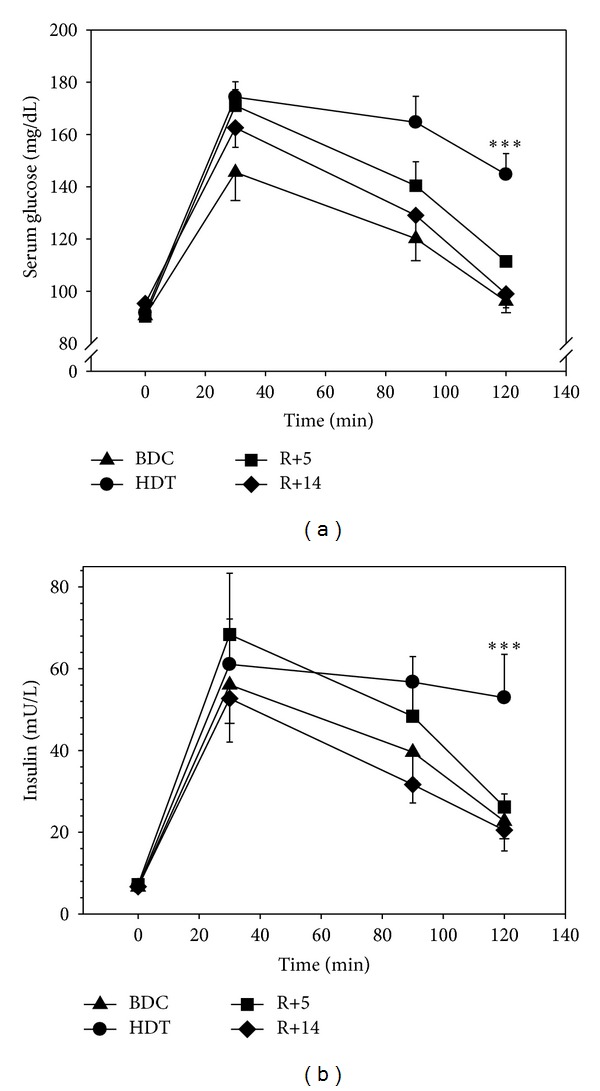
(a) Mean values and SEM of serum glucose concentrations following an oral glucose tolerance test during the in-house ambulatory phase (BDC), on day 21 in head-down tilt bed rest (HDT) and after 4 (R+5, in-house) and 13 (R+14) days of recovery. ***P < 0.001 for the comparison of the prebed rest to bed rest. (b) Mean values of serum insulin concentrations following an oral glucose tolerance test on the identical days and points in time as glucose concentrations were analyzed. ***P < 0.001 for the comparison of the prebed rest to bed rest.

**Figure 2:**
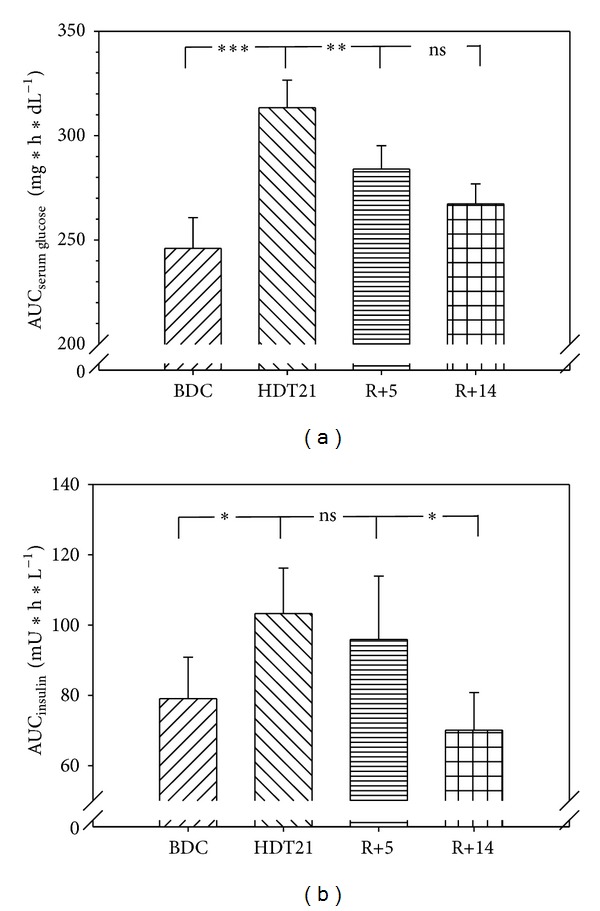
(a) Area under the curve (AUC) of glucose concentrations (mean values (SEM)) after 120 min following an oral glucose tolerance test during the in-house ambulatory phase (BDC), after 20 days (day 21) in head-down tilt bed rest (HDT) and after 4 (R+5, in-house) and 13 (R+14) days of recovery. NS: not significant. *P < 0.05; **P < 0.01. (b) Mean values and SEM of the AUC of serum insulin concentrations (mean values (SEM)) after 120 min following an oral glucose tolerance test on the identical days as glucose concentration was analyzed. NS: not significant. *P < 0.05; **P < 0.01; ***P < 0.001.

Application of potassium bicarbonate during bed rest did not affect glucose and insulin concentrations (data not shown). We therefore based further analyses on the respective mean values of each subject from both bed-rest campaigns. Fasting serum glucose and insulin concentrations were unaffected by bed rest (Figure 1). However, both concentrations were significantly higher in bed rest 120 min after glucose application (both: *P* < 0.001) (Figure 1). On day 21 of bed rest AUC_120 min_ of glucose concentration was significantly increased by 28.8 (5.2)% and AUC_120 min_ of insulin concentrations by 35.9 (10.2)% (glucose: *P* < 0.001; insulin: *P* = 0.02) (Figure 2). While AUC_120 min_ of serum insulin concentrations on R+14 returned to pre-bed-rest concentrations (*P* = 0.352), the respective AUC_120 min_ of glucose was still higher than the pre-bed-rest concentration (*P* = 0.038) (Figure 2).

Insulin resistance analyzed by HOMA-IR2 was not affected by bed rest (Table 1). However, ISI_composite_ index was significantly decreased during bed rest (*P* = 0.002) and returned to pre-bed-rest concentrations on day R+14 (*P* = 0.955) (Table 1). HIR index did not show any effects induced by bed rest (Table 1).

**Figure 3:**
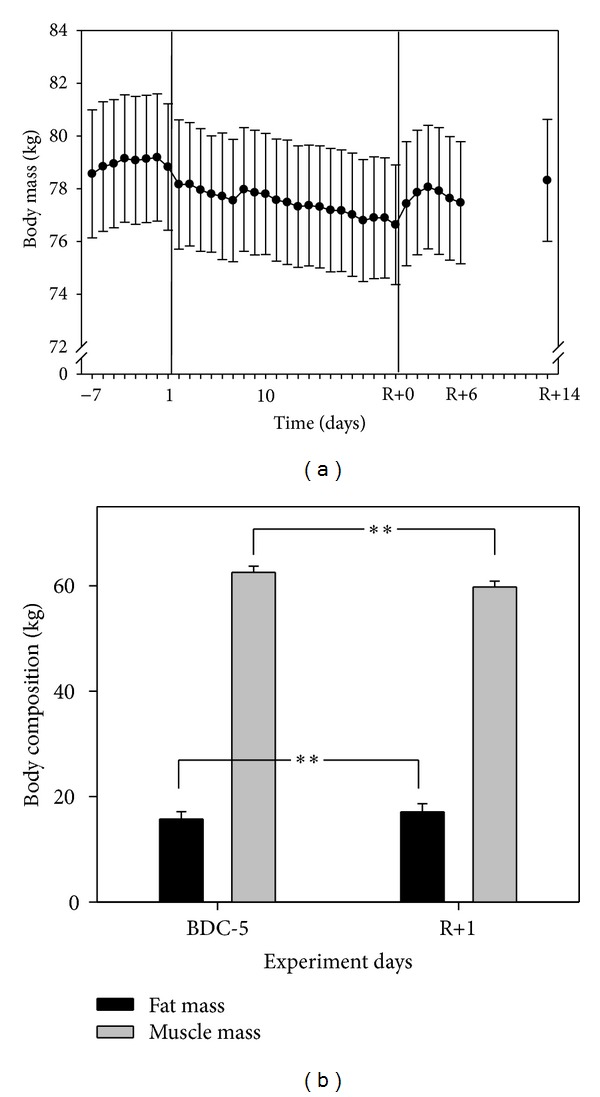
(a) Mean values and SEM of daily body mass data during the entire in-house phase and on day R+14. The vertical lines after day 1 and day R+0 represent the period of HDT bed rest. Body mass decreased significantly during the bed-rest period (*P* < 0.001). (b) Mean values and SD of fat and muscle mass analyzed by DXA 5 days before HDT bed rest and on the first recovery day. **P < 0.01.

Body mass was 1.9 (0.15) kg lower (*P* < 0.001) on day 21 of bed rest compared to prebed rest (Figure 3). However, by day R+6 the difference was decreased to 1.4 (0.15) kg (*P* < 0.001). During the bed rest period fat mass increased by 1.3 (0.3) kg (*P* = 0.002), while muscle mass decreased by 2.8 (0.5) kg (*P* = 0.002).

As expected, CRP concentration increased during bed rest (BDC-2: 0.64 (0.23) mg/L; R+2: 3.85 (0.23) mg/L, *P* < 0.001). Triglyceride and total cholesterol concentrations, however, decreased until day R+2 (triglycerides: *P* < 0.001; total cholesterol: *P* < 0.001) (Table 2), most likely because of changes in dietary composition in the metabolic ward period.

## 4. Discussion

In the presented study we induced impaired glucose tolerance in metabolically healthy, sedentary young male subjects without any first degree relatives being diabetic during experimental bed rest. We could demonstrate that when reassuming the sedentary lifestyle after bed rest four days is insufficient to achieve pre-bed-rest glucose tolerance. When applying a low level of physical activity after bed rest, it takes between five and 14 days even in healthy, nonobese, young subjects to reverse bed-rest induced impaired glucose tolerance. Although the AUC for glucose on day 5 in the recovery phase showed a significant decrease compared to the end of bed rest, the AUC for insulin still demonstrated the compensatory effect.

Since positive energy balance also induced impaired glucose tolerance, we tried to keep fat mass constant by providing individually tailored energy supply. When keeping neutral energy balance by matching energy intake and expenditure and combining the crossover design with our chosen highly standardized study conditions, this led to significantly impaired glucose tolerance in our 7 volunteers. These results are in line with findings from other studies although in those more subjects were needed to clearly show this effect [4, 18–21]. In contrast to several other studies, in our study HOMA-IR was not affected while ISI_composite_ demonstrated significant decreases. HOMA-IR seems to be more influenced by fasting glucose concentrations. Based on experiments by Tripathy et al., it seems that when fasting glucose is only minimally elevated HOMA-IR reflects rather hepatic than peripheral insulin sensitivity [22]. The data would then be in line with HIR and might explain why in the presented bed-rest study we saw a decrease in ISI_composite_ derived from an OGTT but not in HOMA-IR.

One might argue that bed-rest induced increases in fat mass of about 1.3 kg with concomitant decrease in lean body mass (ca. 2.8 kg) might have induced a decrease in insulin sensitivity. We have previously shown [23] that in bed rest volunteers with more pronounced positive energy balance (fat mass increase >1.8 kg) show higher markers of systematic inflammation. This in turn could result in reduced insulin sensitivity. Since six of our seven test subjects had lower fat mass increase than 1.8 kg we feel that—although we should consider it as one cause to increase CRP concentration and in turn reduce insulin sensitivity—this may not be the main cause for the observed effects on insulin sensitivity during bed rest.

After bed rest the subjects got back to ambulatory conditions meaning they were allowed to follow a light physical activity schedule. After the in-house phase, the subjects continued their day-by-day activity, which, as shown by Belavý et al. [17] in the same group, did not differ from pre-bed-rest activity level. However, after a phase of almost enforced inactivity for three weeks, it may take up to two weeks to get back to pre-bed-rest glucose tolerance. These observations are supported by gene expression studies during bed rest carried out by Alibegovic et al. [20]. About 80% of the genes they tested were downregulated and 21% upregulated and of the transcriptional changes they tested only a part was normalized after 4 weeks of retraining. They found changed gene expressions of several insulin resistance and diabetes candidate genes following 9 days of bed rest in their group of similar VO_2_ max as our volunteers. After a four-week retraining period of endurance exercise being cycle ergometry (30 min/day, 6 days/week on 70% of subjects VO_2_ max) a lack of complete normalization of these genes still existed, even though the training led to increased VO_2_ max levels in the volunteers suggesting a more intensified training regime than their usual pre-bed-rest activity level. Hence, although the exercise level was increased, still the gene expression pattern did not revert to the status observed prior to the bed-rest treatment. It might be speculated whether resistive exercise following bed rest would be more effective. Recent evidence has emerged to suggest that even a single bout of resistive exercise improves insulin sensitivity [24–27]. However, further studies are urgently needed to show which kind and intensity of exercise might be able to counteract inactivity induced insulin resistance in healthy subjects.

Our pilot study does have some limitations. Since the primary outcome parameter was the effects of bed rest and potassium bicarbonate supplementation on bone turnover markers, it was not powered to study the post-bed-rest effects on insulin sensitivity, although calculation of post hoc power revealed 100% power for our results in our 7 test subjects. We were not allowed to draw blood according to a usually foreseen schedule when analyzing insulin sensitivity by ISI_composite_. Additionally, no further blood samples or tissue sampling was possible to get further insight into potential mechanisms leading to bed-rest induced insulin resistance.

In summary, our data suggest that it takes between 5 and 14 days for sedentary healthy test subjects to recover from impaired glucose tolerance because of inactivity. When translating these results into clinical conditions where patients usually suffer from comorbidities, further research is urgently mandatory to evaluate a minimum training regime to avoid glucose intolerance merely because of bed-rest induced inactivity.

## Figures and Tables

**Table 1:** Mean values (SEM) of insulin resistance and insulin sensitivity indices.

Day	BDC-4	HDT21	R+5	R+14	*P* value
HOMA IR2	0.86 ( 0.08)	0.88 (0.08)	0.94 (0.09)	0.89 (0.16)	0.790
ISI composite	7.66 ( 1.13)	5.49 (0.60)	6.22 (0.70)	7.59 (0.84)	0.005
HIR	933 (172)	1134 (200)	1236 (250)	933 (151)	0.125

HOMA-IR: homeostatic model assessment-insulin resistance; ISI: insulin sensitivity index [14]; HIR: hepatic insulin resistance index [15].

**Table 2:** Mean values of C-reactive protein, triglycerides, and total cholesterol.

Day	BDC-6	BDC-2	R+2	R+5	*P* value
C-reactive protein (mg/L)	0.89 (0.23)	0.64 (0.23)	3.85 (0.23)*	1.20 (0.23)	<0.001
Triglycerides (mmol/L)	1.41 (0.10)	1.38 (0.10)	1.12 (0.10)*	1.21 (0.10)	<0.001
Total cholesterol (mmol/L)	5.21 (0.05)	4.78 (0.05)	3.89 (0.05)*	4.23 (0.05)	<0.001

*Value is significantly (*P* < 0.001) different from that one on day BDC-2.
